# Editorial: Insights in systems microbiology: 2022/2023

**DOI:** 10.3389/fmicb.2024.1538030

**Published:** 2025-01-14

**Authors:** Qi Zhao, Biswarup Sen, Davida S. Smyth, Fernando Luis Cônsoli

**Affiliations:** ^1^School of Computer Science and Software Engineering, University of Science and Technology Liaoning, Anshan, China; ^2^Wenzhou Institute, University of Chinese Academy of Sciences, Wenzhou, China; ^3^Centre for Marine Environmental Ecology, School of Environmental Science and Engineering, Tianjin University, Tianjin, China; ^4^Department of Natural Sciences, Texas A&M University-San Antonio, San Antonio, TX, United States; ^5^Insect Interactions Laboratory, Department of Entomology and Acarology, Luiz de Queiroz College of Agriculture, University of São Paulo, Piracicaba, São Paulo, Brazil

**Keywords:** systems microbiology, gut microbiome, microorganism, oral microbiota, microbiome diversity

Systems microbiology is a field that plays a pivotal role in deciphering the complexity of microbial life. Studying microorganisms in their natural environments reveals the profound ways in which these organisms contribute to various ecological processes. From nutrient cycling and disease transmission to the synthesis of bioactive compounds, microorganisms play a vital role in sustaining life on Earth. Beyond environmental science, this field holds immense value in healthcare, contributing to the identification of novel pathogens, the development of antibiotics, and a deeper understanding of how the human microbiome impacts health and disease. The multidisciplinary nature of systems microbiology integrates tools and perspectives from genetics, ecology, and bioinformatics, enabling researchers to unravel the complex relationships within microbial communities. The insights gained from this field have far-reaching applications, offering solutions to challenges in agriculture, medicine, and environmental conservation. As our understanding of microbial interactions deepens, the significance of systems microbiology continues to grow, establishing it as a cornerstone of modern scientific research and an essential tool for addressing global challenges.

In this Research Topic, we aim to highlight key advancements and emerging trends in systems microbiology, drawing on recent research conducted between 2022 and 2023. After receiving 16 submissions, six high-quality articles were selected for publication following a rigorous peer-review process.

The Research Topic is particularly notable for its significant contributions to our understanding of the gut microbiome. For example, Huang et al. performed a comprehensive bibliometric analysis using data from the Web of Science Core Collection to chart the evolution of research on the gut microbiome. Their findings highlighted the growing focus on gastrointestinal disorders, particularly conditions such as inflammatory bowel disease, ulcerative colitis, and Crohn's disease. In addition, the impact of gut microbiota on extra-intestinal diseases is gaining recognition, with emerging links to conditions such as obesity, diabetes, cardiovascular diseases, and even neurodegenerative disorders such as Alzheimer's and Parkinson's disease. Lyu et al. employed two-sample Mendelian randomization to uncover microbiota types that influence infection risk. Their research identified 18 microbiota that confer protection against infections and 13 associated with increased susceptibility. Among these, the Ruminococcaceae and Lachnospiraceae families, known for their butyrate production, were found to have both beneficial and detrimental effects on health.

Another key theme explored in this Research Topic is the role of oral microbiota in human health. Bao et al. introduced a novel approach called oral_voting_transfer, designed to improve the classification of oral microbiota species. This model successfully classified organelle proteins across five microorganisms, including *Streptococcus mutans* and *Staphylococcus aureus*. In a related study, Su et al. provided an in-depth analysis of how oral microbiota contribute to cancer development, shedding light on their role in tumorigenic processes and highlighting their potential as biomarkers for early cancer detection and prevention.

Two additional papers further expanded on this Research Topic by exploring microbiome diversity in non-human contexts. Aoki et al. developed a pipeline for analyzing the genomic diversity of the rice rhizosphere microbiome at the single-cell level. This technique enabled them to sequence over 3,000 single-amplified genomes, providing valuable insights into microbial functions within the paddy ecosystem. On the other hand, Soberanes-Gutiérrez et al. constructed a gene co-expression network for *Ustilago maydis*, a pathogenic fungus. Their analysis identified 13 key modules and highlighted critical transcription factors related to its pathogenicity, including those involved in nucleic acid binding, winged helix DNA binding, and the Zn2/Cys6 DNA-binding protein families.

To conclude this editorial, the studies featured in this Research Topic highlight the importance of systems microbiology in addressing key challenges across various domains. Using methods such as bibliometric analysis, Mendelian randomization, single-cell sequencing, and gene co-expression network construction, these studies enhance our understanding of microbial systems and their applications. The findings deepen our knowledge of the gut and oral microbiomes, exploring their roles in health and disease, while also examining microbial diversity in agricultural and pathogenic contexts. These contributions demonstrate how systems microbiology connects basic research with practical applications, driving progress in healthcare, agriculture, and environmental management.

